# Hiding Messages in Secure Connection Transmissions with Full-Duplex Overt Receiver

**DOI:** 10.3390/s22155812

**Published:** 2022-08-03

**Authors:** Lap Luat Nguyen, Tien-Tung Nguyen, Anthony Fiche, Roland Gautier, Hien Q. Ta

**Affiliations:** 1 School of Electrical Engineering, International University, Ho Chi Minh City 700000, Vietnam; nlluat@hcmiu.edu.vn; 2 Vietnam National University, Ho Chi Minh City 700000, Vietnam; 3 Telecommunication Division, Industrial University of Ho Chi Minh City, Ho Chi Minh City 700000, Vietnam; nguyentientung@iuh.edu.vn; 4 Univ Brest, CNRS, Lab-STICC, CS 93837, 6 Avenue Le Gorgeu, CEDEX 3, 29238 Brest, France; anthony.fiche@univ-brest.fr (A.F.); roland.gautier@univ-brest.fr (R.G.)

**Keywords:** covert communication, reliable deniable communication, covert throughput

## Abstract

This paper considers hiding messages in overt transmissions with a full-duplex receiver, which emits artificial noise to secure its transmission connection while a transmitter opportunistically sends a covert message to a covert user. The warden’s uncertainties in decoding the overt message and artificial-noise-received power are exploited to hide messages. Then, the covert throughput accompanied with the warden’s average detection error probability are determined. The results show that increasing the transmit power of artificial noise or improving secure connection at the overt user will improve the covert performance. The results also show that the covert performance is improved when the self-interference cancellation is improved at the full-duplex receiver or when the warden is located close to the full-duplex receiver, indicating the positive impact of the overt performance on the covert performance.

## 1. Introduction

In wireless transmissions, the security and privacy of their broadcast nature become a critical issue as it operates not only in the civil area but also in the military. For example, privacy information in health care, the journey or location of vehicles in transportation and the position or confidential information of the targets need to be protected. Regarding security protection for wireless communications, several conventional approaches, such as cryptography [1] or physical layer security [2,3,4], have been implemented. These methods only target preventing the confidential content of messages from being stolen by eavesdroppers. Nevertheless, some scenarios are dedicated to avoiding being attacked, such as jamming when the existence (privacy) of transmissions is revealed. For example, in the case of a self-driving car, the controlling signals need to be completely secured or hidden from the adversary. Moreover, the location or itinerary of vehicles needs to be kept private. Their revelations to the attacker may cause security issues or accidents. Therefore, covert communication has emerged as a potential solution for dealing with the issue of privacy.

Covert or low-probability-of-detection communication refers to scenarios where the transmitter sends its message to the receiver such that the warden cannot detect (or can detect at a low probability) the existence of transmissions [5]. The square root law (SRL) for covert communication was firstly introduced in additive white Gaussian noise (AWGN) channels for covert or low-probability-of-detection communication with two important measures: the warden’s detection error probability ξ, defined as the sum of the probabilities of a false alarm when the transmitter is not sending and missed the detection when the transmitter is sending, and the covert throughput, defined by the number of bits transmitted reliably over *N* channels subject to the constraint of ξ≥1−ϵ for covert requirement ϵ≥0 [5]. Then, SRL can be used to preside the covert communication, in which, the $O(\sqrt{N})$ bits can be transmitted over *N* channels covertly and reliably. After proving the SRL in additive white Gaussian noise (AWGN) channels [5], it was developed to discrete memoryless channels (DMCs) [6,7,8], in which, the Big-O notation characterizes the constant hidden. Then, [9] extended the study to covert communication in DMCs under some constraints related to the covertness. In [10], the authors showed that the Gaussian signalling can be optimal under only a covertness constraint. Thus, the Kullback–Leibler divergence asymmetry property can optimize the Gaussian signalling with other constraints to [10]. However, SRL has a weakness, as a positive covert rate cannot be guaranteed when the number of channels tends to infinity. This means that the covert rate O(N) tends to 0 when *N* tends to infinity. This problem of SRL was later addressed and solved by [11,12,13]. In [11], the study pointed out that a signal-to-noise ratio (SNR) threshold will impact the detection of the transmitted signal if the received signal power is less than this threshold. Thus, it can be seen as a noise uncertainty when the noise power estimation does not match the actual noise power. Hence, the noise uncertainty is useful in covert communication since it could be a medium to hide a message. The study in [12] proved that there will be a positive covert rate even for an infinite number of channels if the legitimate has noise uncertainty. Thus, the covert throughput was analyzed in [13] based on two noise uncertainty practical models under AWGN channels.

In general, two well-known approaches adopted for covert communications have been widely investigated in the literature. The key idea of the first approach is to take advantage of various sources of the adversary’s uncertainty to guarantee the covertness, e.g., adversary’s noise uncertainty in [12,13], uncertainties of transmission time [14], channel state information [15] and transmit power [16]. Later, the noise uncertainty was extended to uninformed jamming [17] and artificial noise (AN) from a friendly jammer [18] or from a full-duplex receiver [19,20,21]. Using AN at the FD receiver, the receiver receives the covert message while jamming the warden to make it harder to detect the covert message [19,20,21]. However, two critical questions arise: (1) in the case of the covert receiver being a low cost-device, i.e., IoT users with hardware that is not complex and does not have enough energy to simultaneously carry out two such roles, and (2) jamming by the covert receiver, which receives the covert message, may help the adversary to detect the presence of covert transmission. Hence, jamming while receiving the covert information by the covert receiver may not be practical in a real scenario. For the second approach, other works considered superimposing the covert message into another message of existing transmissions, termed as hiding messages into overt transmissions, and exploited transmit power control [22] and random transmit power [23]. The basic idea of the second approach is to guarantee covertness by enlarging the dynamic range of the adversary’s uncertainties via the existing transmission. Most recently, [24] showed, for the first time, that the warden cannot detect the covert message if the overt message where the covert message is superimposed onto is not decoded. This finding can be referred to as *decoding uncertainty* at the warden.

Inspired by [24] and the full-duplex receiver scheme in [19,20,21], we introduce a novel scheme of hiding messages into existing overt transmissions with a FD overt receiver. Different from [19,20,21], the artificial noise jamming signal is transmitted by the overt receiver with just naive intention to secure its connection with the transmitter [25] and, thus, the presence of the covert transmission will not be detected via artificial noise jamming. More specifically, the superposition signal of the covert and overt messages is transmitted at a fixed transmit power while the overt receiver emits the AN to secure its connection. With this setting, we find that, in order to detect the covert message, it is necessary for the warden to decode the overt message and, even if the overt message is decoded, the covertness still can be guaranteed due to the adversary’s uncertainty in receiving AN powers. Then, an efficient AN and the improved performance of the secure connection of the overt transmission leads to an improvement in the covertness. In this paper, performances were evaluated by using three metrics: the adversary’s average detection error probability ξ, the covert throughput $\eta_{u}$ and the overt throughput loss $\eta_{v,\mathrm{loss}}$. The main contributions of the paper are listed as follows:

- We propose a scheme to hide the covert information by exploiting the existing secure connection transmission with the aid of artificial noise generated by a full-duplex overt receiver, which has not been considered in literature.
- The artificial noise used for improving the secure connection transmission can also be exploited for improving covert transmissions.
- The artificial noise can be further improved if the channel state information between the over receiver and the warden is unknown at the warden, which is also known as the uncertainty of artificial noise power.

This paper is organized as follows. Section 2 will describe the system model with one transmitter and two receivers, as well as a warden. Section 3 presents the optimum detection of the warden. The covert throughput and the overt throughput loss are examined in Section 4 and Section 5, respectively. The numerical results will be shown in Section 6.

## 2. System Model

**Figure 1:**
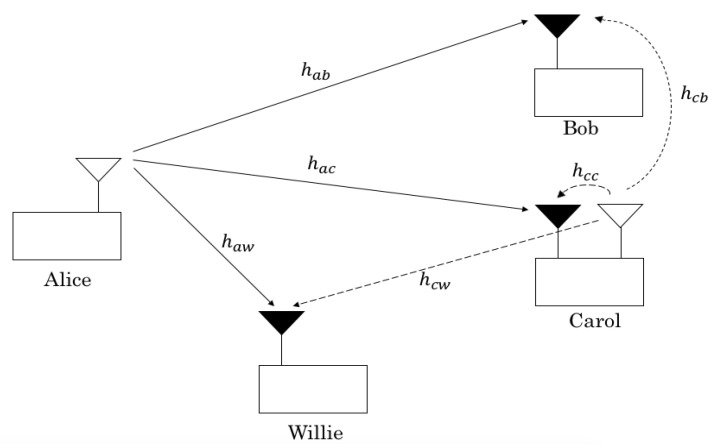
Alice tries to hide a covert message to Bob within overt transmissions to Carol toward Willie; a warden looks for covert message.

We considered one transmitter (Alice) and two receivers—one (Carol) to receive overt messages, **v** = $\left(v_{1},...,v_{n}\right)$, and one (Bob) to receive covert messages, **u** = $\left(u_{1},...,u_{n}\right)$—and a warden (Willie) to detect the presence of covert transmission. The proposed system is illustrated in Figure 1, where Carol equipped with two antennas operates at full-duplex mode to secure its connection to the warden [19], whereas other nodes employ a half-duplex mode with a single antenna. The proposed scheme is under practical consideration of uplink transmissions, in which, the transmitter (an IoT device) opportunistically sends its covert message on top of the overt message as a camouflage while the overt receiver (a base station with an advanced receiver architecture) secures its connection. Here, the artificial noise is naively exploited to secure the connection for the overt user and, then, is opportunistically used to provide covertness for the covert user.

Considering the random coding used to generate codewords [26], the codewords u and v are independently generated by random selection symbols from a complex normal distribution. To guarantee the covertness, the codebook of u is a share secret between Alice and Bob, whereas that of v is assumed as known to all users, including Willie. The transmitted signal from Alice, from Willie’s perspective, is given by

(1) $$\begin{array}{c} x=\left\{\begin{array}{cc} \sqrt{P_{a}}v,\;\;\;H_{0}, & \\ \sqrt{P_{a}}\left(\sqrt{\alpha }v+\sqrt{1-\alpha }u\right),\;H_{1}, & \end{array}\right. \end{array}$$

where $H_{0}$ and $H_{1}$ denote the null hypothesis that u has not been sent by Alice and the alternative hypothesis, respectively, α∈[0,1] is the ratio of power allocated to the overt message and $P_{a}$ is Alice’s total transmit power, which is always constant regardless of the covert transmission.

The quasi-static Rayleigh block fading channels is considered, where the channel gain is constant inside an *n* symbols block and changes from one block to another independently [22,24]. Let the channel gain be $h_{ij}$ between nodes *i* and *j*, where i∈{a,c} and j∈{b,c,w}, in which nodes a,b,c and *w* represent Alice, Bob, Carol and Willie, respectively, and have a Gaussian distribution with mean 0 and variance $\sigma_{ij}^{2}$. A high value of $\sigma_{ij}^{2}$ means that the two users are close. Assuming that Alice sends pilot symbols for channel estimations before data transmissions, and assuming perfect channel estimation at all receiver nodes, node *j* perfectly knows $h_{aj}$. We further denote $n_{j}$, j∈{b,c,w} as the background noise vector at node *j* and assume that $n_{j}$ has a complex Gaussian distribution with mean 0 and variance $\sigma_{n}^{2}$, i.e., $n_{j}∼CN\left(0,\sigma_{n}^{2}\right)$. It should be noted that Carol does not send their channel estimate to Alice. In addition, Bob will not send their channel estimate to Alice in order to avoid being detected by Willie. Hence, Alice does not know the channel information from them to other nodes and designs fixed transmission rates to Bob and Carol. This design is suitable for IoT applications with a requirement of strict latency as it does not require channel information feedback. For convenience, the parameter and metric notations are provided in Table 1.

## 3. Warden’s Optimum Detection

After receiving the transmitted signal from Alice and artificial noise from Carol, the signal to be received at Willie is expressed as

(2) $$y_{w}=\left\{\;\begin{array}{cc} \sqrt{P_{a}}h_{aw}v+\sqrt{P_{c}}h_{cw}z+n_{w},\;\;\;H_{0}, & \\ \sqrt{P_{a}}h_{aw}\left(\sqrt{\alpha }v+\sqrt{1-\alpha }u\right)+\sqrt{P_{c}}h_{cw}z+n_{w},\;H_{1}, \end{array}\right.$$

where z∼CN(0,1) is the AN signal transmitted by Carol and $P_{c}$ is the transmit AN power. It will be proven that the distribution of $y_{w}=\left\{y_{w,1},\cdots ,y_{w,n}\right\}$ is identical under $H_{0}$ and $H_{1}$ if v is not decoded, and different if v is decoded. This means that Willie cannot detect the covert message u if the overt message v cannot be decoded. Moreover, since Willie has uncertainty in the received AN power, $|h_{cw}{|}^{2}P_{c}$, the covertness can still be guaranteed if v is decoded. In this section, the average total detection error probability, estimated over the event of Willie’s success and failure to decode v, is calculated. In detail, we considered two cases: Willie fails to decode v and Willie succeeds in decoding v, as follows.

### 3.1. Willie Fails to Decode v

When Willie fails to decode v, they will perform the marginalized likelihood ratio test (LRT), i.e., averaging unknown v in the likelihood functions, as its optimum detection [27], is

(3) $$\Lambda :=\frac{E_{v}\left[\mathrm{Pr}\left(y_{w}|v,H_{1}\right)\right]}{E_{v}\left[\mathrm{Pr}\left(y_{w}|v,H_{0}\right)\right]}\underset{H_{1}}{\overset{H_{0}}{⋚}}\lambda ,$$

where ($E_{X}\left[f\left(X\right)\right]$ denotes the expectation of the function f(X) with respect to the random variable *X*), under $H_{1}$, by treating $u=\{u_{1},...,u_{n}\}$ in (2) as noise since Willie does not know the codebook of u, we have

(4) $$\begin{array}{ccc} \;\mathrm{Pr}(y_{w}|v,H_{1})\; & \;=\; & \;\prod_{i=1}^{n}\mathrm{Pr}\left(y_{w,i}|v_{i},H_{1}\right)\\ \; & \;=\; & \;\prod_{i=1}^{n}\frac{\exp\left(-\frac{|y_{w,i}-h_{aw}\sqrt{\alpha P_{a}}v_{i}{|}^{2}}{\sigma_{n}^{2}+|h_{cw}{|}^{2}P_{c}+\left(1-\alpha \right)P_{a}{\left|h_{aw}\right|}^{2}}\right)}{\pi (\sigma_{n}^{2}+|h_{cw}{|}^{2}P_{c}+\left(1-\alpha \right){\left|h_{aw}\right|}^{2}P_{a})}\\ \; & \;=\; & \;\frac{\exp\left(-\frac{{∥y_{w}-h_{aw}\sqrt{\alpha P_{a}}v∥}^{2}}{\sigma_{n}^{2}+|h_{cw}{|}^{2}P_{c}+\left(1-\alpha \right)P_{a}{\left|h_{aw}\right|}^{2}}\right)}{(\pi (\sigma_{n}^{2}+|h_{cw}{|}^{2}P_{c}+\left(1-\alpha \right){\left|h_{aw}\right|}^{2}P_{a}{))}^{n}}, \end{array}$$

and, under $H_{0}$, we similarly have

(5) $$\begin{array}{ccc} \;\mathrm{Pr}(y_{w}|v,H_{0})\; & \;=\; & \;\frac{\exp\left(-\frac{\left|\right|y_{w}-h_{aw}\sqrt{P_{a}}v{\left|\right|}^{2}}{\sigma_{n}^{2}+{\left|h_{cw}\right|}^{2}P_{c}}\right)}{(\pi (\sigma_{n}^{2}+|h_{cw}{|}^{2}P_{c}{))}^{n}}. \end{array}$$

Then, we obtain from (4) and (5) that

(6) $$E_{v}\left[\mathrm{Pr}\left(y_{w}|v,H_{0}\right)\right]=E_{v}\left[\mathrm{Pr}\left(y_{w}|v,H_{1}\right)\right].$$

The proof of (6) is provided in Appendix A. Hence, Λ=1.

Let $I(v;y_{w})$ denote the mutual information between v and $y_{w}$, and $R_{v}$ denote the transmission rate of the overt message. The design value of $R_{v}$ will be determined in Section 5. It follows from [28] (Equation (1)) that the event of $I\left(v;y_{w}\right)<R_{v}$ is a subset of the event that Willie fails to decode v. Then, the lower bound of the false alarm and missed detection probabilities, in the case that Willie fails to decode v, are given by

(7) $$\begin{array}{ccc} P_{f} & \;=\; & \mathrm{Pr}(\lambda <1,I\left(v;y_{w}\right)<R_{v}|H_{0}),\\ P_{m} & \;=\; & \mathrm{Pr}(\lambda \geq 1,I\left(v;y_{w}\right)<R_{v}|H_{1}), \end{array}$$

respectively, where

(8) $$I\left(v;y_{w}\right)=\left\{\begin{array}{cc} \log_{2}\left(1+\frac{|h_{aw}{|}^{2}P_{a}}{\sigma_{n}^{2}+{\left|h_{cw}\right|}^{2}P_{c}}\right),\;H_{0}, & \\ \log_{2}\left(1+\frac{\alpha |h_{aw}{|}^{2}P_{a}}{\sigma_{n}^{2}+\left(1-\alpha \right)|h_{aw}{|}^{2}P_{a}+{\left|h_{cw}\right|}^{2}P_{c}}\right),\;H_{1}. \end{array}\right.$$

To achieve a high detection performance, the strategy of the warden Willie is to minimize the sum of the false alarm and missed detection probabilities ($P_{f}+P_{m}$ ) by choosing λ properly. Since $I(v;y_{w})$ under $H_{0}$ is larger than that under $H_{1}$, Willie may choose λ<1 to obtain the minimum

(9) $$\begin{array}{cc} \;P_{f}+P_{m} & =\mathrm{Pr}(I\left(v;y_{w}\right)<R_{v}|H_{0})\\ & =1-\mathrm{Pr}\left(|h_{aw}{|}^{2}\geq \frac{\left(2^{R_{v}}-1\right)(\sigma_{n}^{2}+|h_{cw}{|}^{2}P_{c})}{P_{a}}\right), \end{array}$$

which, since $|h_{ij}{|}^{2}$ is distributed exponentially with a scale of $1/\sigma_{ij}^{2}$, yields

(10) $$\begin{array}{cc} \;P_{f}+P_{m} & =1-\int_{0}^{\infty }\exp\left(-\frac{\left(2^{R_{v}}-1\right)\left(\sigma_{n}^{2}+xP_{c}\right)}{\sigma_{aw}^{2}P_{a}}\right)\times \frac{\exp(-x/\sigma_{cw}^{2})}{\sigma_{cw}^{2}}dx\\ \; & =1-\frac{\exp(-\left(2^{R_{v}}-1\right)\sigma_{n}^{2}/\left(\sigma_{aw}^{2}P_{a}\right))}{1+\left(2^{R_{v}}-1\right)\sigma_{cw}^{2}P_{c}/\left(\sigma_{aw}^{2}P_{a}\right)}. \end{array}$$

**Remark** **1.** *One can see from (9) that $\left(P_{f}+P_{m}\right)$ also represents the secure connection probability and increases as the AN transmit power $P_{c}$ increases. This means that the AN helps to not only secure the connection for the overt user but also to hide the message for the covert user.*

### 3.2. Willie Succeeds in Decoding v

When Willie succeeds in decoding v, they will perform the LRT of

(11) $$\begin{array}{ccc} \Lambda^{\prime }\; & := & \;\frac{1}{n}\ln\left(\frac{\mathrm{Pr}(y_{w}|v,H_{1})}{\mathrm{Pr}(y_{w}|v,H_{0})}\right)-\ln\left(\frac{\sigma_{n}^{2}+{\left|h_{cw}\right|}^{2}P_{c}}{\sigma_{n}^{2}+|h_{cw}{|}^{2}P_{c}+\left(1-\alpha \right){\left|h_{aw}\right|}^{2}P_{a}}\right)\\ \; & \;=\; & \;\frac{\left|\right|y_{w}-h_{aw}\sqrt{P_{a}}v{\left|\right|}^{2}}{n(\sigma_{n}^{2}+|h_{cw}{|}^{2}P_{c})}-\frac{\left|\right|y_{w}-h_{aw}\sqrt{\alpha P_{a}}v{\left|\right|}^{2}}{n(\sigma_{n}^{2}+|h_{cw}{|}^{2}P_{c}+\left(1-\alpha \right)|h_{aw}{|}^{2}P_{a})}\underset{H_{1}}{\overset{H_{0}}{⋚}}\lambda^{\prime }, \end{array}$$

where (11) is derived from (4) and (5). As n→∞ (the best scenario for Willie’s detection), $\Lambda^{\prime }$ converges to

(12) $$\begin{array}{ccc} \Lambda^{\prime }\; & \to & \;\left\{\begin{array}{cc} \frac{2\sqrt{\alpha }\left(1-\sqrt{\alpha }\right){\left|h_{aw}\right|}^{2}P_{a}}{\sigma_{n}^{2}+|h_{cw}{|}^{2}P_{c}+\left(1-\alpha \right){\left|h_{aw}\right|}^{2}P_{a}},\;\;H_{0}, & \\ \frac{2\left(1-\sqrt{\alpha }\right){\left|h_{aw}\right|}^{2}P_{a}}{\sigma_{n}^{2}+{\left|h_{cw}\right|}^{2}P_{c}},\;H_{1}. \end{array}\right. \end{array}$$

**Remark** **2.** *One can see that, if Willie perfectly knows the channel gain $h_{cw}$ via channel information feedback at Carol or perfectly knows their received AN power of $|h_{cw}{|}^{2}P_{c}$, Willie can choose the detection threshold*

(13) $$\begin{array}{c} \lambda^{\prime }\in \left[\frac{2\sqrt{\alpha }\left(1-\sqrt{\alpha }\right){\left|h_{aw}\right|}^{2}P_{a}}{\sigma_{n}^{2}+|h_{cw}{|}^{2}P_{c}+\left(1-\alpha \right){\left|h_{aw}\right|}^{2}P_{a}},\frac{2\left(1-\sqrt{\alpha }\right){\left|h_{aw}\right|}^{2}P_{a}}{\sigma_{n}^{2}+{\left|h_{cw}\right|}^{2}P_{c}}\right] \end{array}$$

*such that, from (12), the false alarm probability and missed detection probability are zero, i.e., $P_{f}^{\prime }\left(h_{aw}\right)=P_{m}^{\prime }\left(h_{aw}\right)=0$, i.e., Willie can always detect the presence of the covert transmission if the overt message v is decoded and removed. However, this channel information feedback is not considered in our system model and, hence, Willie has uncertainty in the received AN power, which still guarantees a certain covertness even if the overt message v is decoded and removed.*

Since $|h_{cw}{|}^{2}$ has an exponential distribution with a mean of $\sigma_{cw}^{2}$, the false alarm and missed detection probabilities for decoding u at Willie, by assuming that Willie succeeds in decoding v, based on (8) and (12), are given by

(14) $$\begin{array}{ccc} \;P_{f}^{\prime }\left(h_{aw}\right) & \;=\; & \;\mathrm{Pr}(\Lambda^{\prime }>\lambda^{\prime },I\left(v;y_{w}\right)\geq R_{v}|H_{0})\\ \; & \;=\; & \;\mathrm{Pr}(|h_{cw}{|}^{2}/\sigma_{cw}^{2}<\min\left\{r_{0}/\lambda^{\prime }-s_{0},\delta_{0}\right\})\\ \; & \;=\; & \;1-\exp\left(-\min\{r_{0}/\lambda^{\prime }-s_{0},\delta_{0}\}\right), \end{array}$$

(15) $$\begin{array}{ccc} \;P_{m}^{\prime }\left(h_{aw}\right)\; & \;=\; & \;\mathrm{Pr}(\Lambda^{\prime }\leq \lambda^{\prime },I\left(v;y_{w}\right)\geq R_{v}|H_{1})\\ \; & \;=\; & \;\mathrm{Pr}\left(r_{1}/\lambda^{\prime }-s_{1}\leq {\left|h_{cw}\right|}^{2}/\sigma_{cw}^{2}\leq \delta_{1}\right)\\ \; & \;=\; & \;{\left(\exp\left(-\left(r_{1}/\lambda^{\prime }-s_{1}\right)\right)-\exp\left(-\delta_{1}\right)\right)}^{+}, \end{array}$$

where ${\left(x\right)}^{+}=\max\left(x,0\right)$, and

(16) $$\begin{array}{ccc} \;r_{0}\; & \;=\; & \;2\sqrt{\alpha }\left(1-\sqrt{\alpha }\right){\left|h_{aw}\right|}^{2}P_{a}/\left(\sigma_{cw}^{2}P_{c}\right),\\ \;s_{0}\; & \;=\; & \;(\sigma_{n}^{2}+\left(1-\alpha \right)|h_{aw}{|}^{2}P_{a})/\left(\sigma_{cw}^{2}P_{c}\right),\\ \;r_{1}\; & \;=\; & \;2\left(1-\sqrt{\alpha }\right){\left|h_{aw}\right|}^{2}P_{a}/\left(\sigma_{cw}^{2}P_{c}\right),\\ \;s_{1}\; & \;=\; & \;\sigma_{n}^{2}/\left(\sigma_{cw}^{2}P_{c}\right),\\ \;\delta_{0}\; & \;=\; & \;|h_{aw}{|}^{2}P_{a}/\left(\left(2^{R_{v}}-1\right)\sigma_{cw}^{2}P_{c}\right)-\sigma_{n}^{2}/\left(\sigma_{cw}^{2}P_{c}\right),\\ \;\delta_{1}\; & \;=\; & \;|h_{aw}{|}^{2}P_{a}{\left(\alpha \left(1-\alpha \right)2^{R_{v}}\right)}^{+}/\left(\left(2^{R_{v}}-1\right)\sigma_{cw}^{2}P_{c}\right)-\sigma_{n}^{2}/\left(\sigma_{cw}^{2}P_{c}\right). \end{array}$$

Willie attempts to minimize $\left(P_{f}^{\prime }\left(h_{aw}\right)+P_{m}^{\prime }\left(h_{aw}\right)\right)$ by properly choosing $\lambda^{\prime }$ equal to

(17) $$\begin{array}{ccc} \lambda^{\prime }\; & \;=\; & \;{\left[\min\left\{\frac{\ln\left(r_{1}/r_{0}\right)-\left(s_{0}-s_{1}\right)}{r_{1}-r_{0}},\frac{s_{1}+\delta_{1}}{r_{1}}\right\}\right]}^{-1}, \end{array}$$

where its proof is provided in Appendix B, and then

(18) $$\begin{array}{ccc} P_{f}^{\prime }\left(h_{aw}\right)+P_{m}^{\prime }\left(h_{aw}\right)\; & \;=\; & \;1-\exp\left(s_{0}-r_{0}/\lambda^{\prime }\right)+\exp\left(s_{1}-r_{1}/\lambda^{\prime }\right))-\exp\left(-\delta_{1}\right). \end{array}$$

### 3.3. Average Total Detection Error Probability

In summary, the average total detection error probability, estimated over the event of Willie’s success and failure to decode v regardless of the number of symbols *n*, can be determined as

(19) $$\begin{array}{ccc} \xi \; & \;=\; & \;\left(P_{f}+P_{m}\right)+E_{h_{aw}}\left[P_{f}^{\prime }\left(h_{aw}\right)+P_{m}^{\prime }\left(h_{aw}\right)\right], \end{array}$$

which can be computed from (10) and (18). Here, we emphasize that the total detection error probability in (19) is the lower bound obtained from (10) and (18) and that it is necessary to consider (19) as the covertness measure, which is independent of the number of symbols under practical considerations.

**Covert requirement:** A covert communication can be achieved if ξ≥1−ϵ for any covertness requirement ϵ>0. This means that the detection is ineffective, i.e., ξ→1, at a sufficiently small ϵ→0.

## 4. Covert Throughput at the Covert User Bob

In this section, the covert throughput will be determined. The covert throughput of u between Alice and Bob is the average rate correctly received over many transmission bursts, i.e., $R_{u}\times \left(1-P_{\mathrm{out},B}\right)$, because the message is only correctly received on $\left(1-P_{\mathrm{out},B}\right)$ transmissions [15], with $P_{\mathrm{out},B}$ being the decoding outage probability. Since Alice does not know $h_{ab}$, they will transmit u at a fixed rate $R_{u}$. The received signal at Bob is given by

(20) $$y_{b}=\sqrt{\left(1-\alpha \right)P_{a}}h_{ab}u+\sqrt{\alpha P_{a}}h_{ab}v+\sqrt{P_{c}}h_{cb}z+n_{b}.$$

Assuming successive interference cancellation receiver type at Bob [29], the covert message maximum rate is given by

(21) $$I{\left(u;y_{b}\right)}_{0}=\log_{2}\left(1+\frac{\left(1-\alpha \right)|h_{ab}{|}^{2}P_{a}}{\sigma_{n}^{2}+\alpha |h_{ab}{|}^{2}P_{a}+{\left|h_{cb}\right|}^{2}P_{c}}\right)$$

if Bob cannot decode v, i.e., $I\left(v;y_{b}\right)<R_{v}$ or, equivalently,

(22) $$|h_{ab}{|}^{2}<\frac{\left(2^{R_{v}}-1\right)(\sigma_{n}^{2}+|h_{cb}{|}^{2}P_{c})}{{\left(\alpha -\left(1-\alpha \right)\left(2^{R_{v}}-1\right)\right)}^{+}P_{a}},$$

and, otherwise,

(23) $$I{\left(u;y_{b}\right)}_{1}=\log_{2}\left(1+\frac{\left(1-\alpha \right)|h_{ab}{|}^{2}P_{a}}{\sigma_{n}^{2}+{\left|h_{cb}\right|}^{2}P_{c}}\right).$$

Since $|h_{ab}{|}^{2}$ has an exponential distribution with a mean of $\sigma_{ab}^{2}$ with its cumulative distribution function (CDF) of

(24) $$\mathrm{Pr}(|h_{ab}{|}^{2}<x)=1-\exp\left(-x/\sigma_{ab}^{2}\right)$$

and $|h_{cb}{|}^{2}$ has an exponential distribution with a mean of $\sigma_{cb}^{2}$ with its probability density function (PDF) of

(25) $$p_{|h_{cb}{|}^{2}}\left(x\right)=\exp\left(-x/\sigma_{cb}^{2}\right)/\sigma_{cb}^{2}.$$

The decoding outage probability, denoted as $P_{\mathrm{out},B}\left(P_{a}\right)$, of the covert message u can be derived in a closed-form expression as

(26) $$\begin{array}{ccc} \;P_{\mathrm{out},B}\left(P_{a}\right)\; & \;=\; & \;\mathrm{Pr}\left(I{\left(u;y_{b}\right)}_{0}<R_{u},{\left|h_{ab}\right|}^{2}<\frac{\left(2^{R_{v}}-1\right)(\sigma_{n}^{2}+|h_{cb}{|}^{2}P_{c})}{{\left(\alpha -\left(1-\alpha \right)\left(2^{R_{v}}-1\right)\right)}^{+}P_{a}}\right)\\ \; & & \;+\;\mathrm{Pr}\left(I{\left(u;y_{b}\right)}_{1}<R_{u},{\left|h_{ab}\right|}^{2}\geq \frac{\left(2^{R_{v}}-1\right)(\sigma_{n}^{2}+|h_{cb}{|}^{2}P_{c})}{{\left(\alpha -\left(1-\alpha \right)\left(2^{R_{v}}-1\right)\right)}^{+}P_{a}}\right)\\ \; & \;=\; & \;\mathrm{Pr}\left(|h_{ab}{|}^{2}<\min\left\{\frac{\left(2^{R_{v}}-1\right)(\sigma_{n}^{2}+|h_{cb}{|}^{2}P_{c})}{{\left(\alpha -\left(1-\alpha \right)\left(2^{R_{v}}-1\right)\right)}^{+}P_{a}},\frac{\left(2^{R_{u}}-1\right)(\sigma_{n}^{2}+|h_{cb}{|}^{2}P_{c})}{{\left(1-\alpha 2^{R_{u}}\right)}^{+}P_{a}}\right\}\right)\\ \; & & \;+\;\mathrm{Pr}\left(\frac{\left(2^{R_{v}}-1\right)(\sigma_{n}^{2}+|h_{cb}{|}^{2}P_{c})}{{\left(\alpha -\left(1-\alpha \right)\left(2^{R_{v}}-1\right)\right)}^{+}P_{a}}\leq {\left|h_{ab}\right|}^{2}\leq \frac{\left(2^{R_{u}}-1\right)(\sigma_{n}^{2}+|h_{cb}{|}^{2}P_{c})}{\left(1-\alpha \right)P_{a}}\right)\\ \; & \;=\; & \;1-\int_{0}^{\infty }\exp\left(-\min\left\{\frac{\left(2^{R_{v}}-1\right)\left(\sigma_{n}^{2}+xP_{c}\right)}{{\left(\alpha -\left(1-\alpha \right)\left(2^{R_{v}}-1\right)\right)}^{+}\sigma_{ab}^{2}P_{a}},\frac{\left(2^{R_{u}}-1\right)\left(\sigma_{n}^{2}+xP_{c}\right)}{{\left(1-\alpha 2^{R_{u}}\right)}^{+}\sigma_{ab}^{2}P_{a}}\right\}\right)\\ \; & & \;\times p_{|h_{cb}{|}^{2}}\left(x\right)dx\\ \; & & \;+\;\int_{0}^{\infty }(\exp\left(-\frac{\left(2^{R_{v}}-1\right)(\sigma_{n}^{2}+|h_{cb}{|}^{2}P_{c})}{{\left(\alpha -\left(1-\alpha \right)\left(2^{R_{v}}-1\right)\right)}^{+}\sigma_{ab}^{2}P_{a}}\right)\\ \; & & \;-\;\exp\left(-\frac{\left(2^{R_{u}}-1\right)(\sigma_{n}^{2}+|h_{cb}{|}^{2}P_{c})}{\left(1-\alpha \right)\sigma_{ab}^{2}P_{a}}\right))p_{|h_{cb}{|}^{2}}\left(x\right)dx\\ \; & \;=\; & \;1-\max\left\{\frac{e^{-\frac{\left(2^{R_{v}}-1\right)\sigma_{n}^{2}}{{\left(\alpha -\left(1-\alpha \right)\left(2^{R_{v}}-1\right)\right)}^{+}\sigma_{ab}^{2}P_{a}}}}{1+\frac{\left(2^{R_{v}}-1\right)\sigma_{cb}^{2}P_{c}}{{\left(\alpha -\left(1-\alpha \right)\left(2^{R_{v}}-1\right)\right)}^{+}\sigma_{ab}^{2}P_{a}}},\frac{e^{-\frac{\left(2^{R_{u}}-1\right)\sigma_{n}^{2}}{{\left(1-\alpha 2^{R_{u}}\right)}^{+}\sigma_{ab}^{2}P_{a}}}}{1+\frac{\left(2^{R_{u}}-1\right)\sigma_{cb}^{2}P_{c}}{{\left(1-\alpha 2^{R_{u}}\right)}^{+}\sigma_{ab}^{2}P_{a}}}\right\}\\ \; & & \;+\;{\left(\frac{e^{-\frac{\left(2^{R_{v}}-1\right)\sigma_{n}^{2}}{{\left(\alpha -\left(1-\alpha \right)\left(2^{R_{v}}-1\right)\right)}^{+}\sigma_{ab}^{2}P_{a}}}}{1+\frac{\left(2^{R_{v}}-1\right)\sigma_{cb}^{2}P_{c}}{{\left(\alpha -\left(1-\alpha \right)\left(2^{R_{v}}-1\right)\right)}^{+}\sigma_{ab}^{2}P_{a}}}-\frac{e^{-\frac{\left(2^{R_{u}}-1\right)\sigma_{n}^{2}}{\sigma_{ab}^{2}P_{a}}}}{1+\frac{\left(2^{R_{u}}-1\right)\sigma_{cb}^{2}P_{c}}{\sigma_{ab}^{2}P_{a}}}\right)}^{+}. \end{array}$$

**Figure 2:**
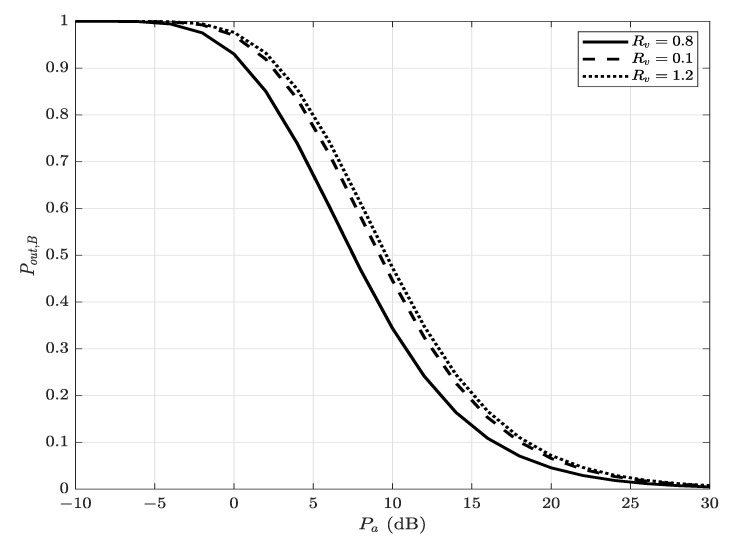
Bob’s decoding outage probability, $P_{\mathrm{out},B}\left(P_{a}\right)$, versus Alice’s transmit power $P_{a}$, for different values of $R_{v}$; α=0.8 and $P_{c}=5$ dB.

**Figure 3:**
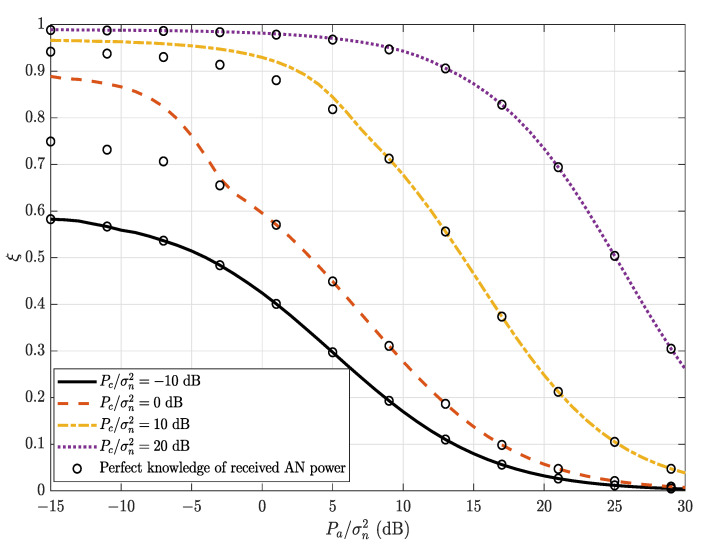
The average detection error probability, ξ, versus Alice’s transmit power, $P_{a}/\sigma_{n}^{2}$, for different values of $P_{c}/\sigma_{n}^{2}$; α=0.8 and ϕ=0.01.

As in [24], the covert throughput (bits/Hz/s) is expressed as $R_{u}\times \left(1-P_{\mathrm{out},B}\left(P_{a}\right)\right)$. In this paper, the covert throughput maximization problem is formulated as

(27) $$\begin{array}{ccc} \eta_{u}=\; & \max_{P_{a}} & R_{u}\times \left(1-P_{\mathrm{out},B}\left(P_{a}\right)\right)\\ \; & s.t. & \xi \geq 1-\epsilon , \end{array}$$

where ϵ represents the covertness requirement. Since $P_{\mathrm{out},B}\left(P_{a}\right)$ is a decreasing function of $P_{a}$ (see Figure 2), $R_{u}\left(1-P_{\mathrm{out},B}\left(P_{a}\right)\right)$ is an increasing function of $P_{a}$. Also, since ξ is a decreasing function of $P_{a}$ (see Figure 3), the constraint of ξ≥1−ϵ requires $P_{a}$ less than a threshold $P_{a}^{*}$, where $P_{a}^{*}$ is the solution of ξ=1−ϵ. Hence, $R_{u}\left(1-P_{\mathrm{out},B}\left(P_{a}\right)\right)$ is maximized when $P_{a}=P_{a}^{*}$. Therefore, the covert throughput (bits/Hz/s) is given by

(28) $$\eta_{u}=R_{u}\times \left(1-P_{\mathrm{out},B}\left(P_{a}^{*}\right)\right).$$

and its resulting maximum covert throughput is given by

(29) $$\eta_{u,max}=\max_{R_{u}}\;\eta_{u}$$

To enable a positive covert throughput, the overt user needs to sacrifice its throughput for the covert message. Consequently, the next section presents the overt throughput loss.

## 5. Overt Throughput Loss at the Overt User Carol

In this section, the loss of the overt throughput traded for the covert throughput is characterized. Under $H_{1}$, the received signal at Carol is given by

(30) $$y_{c}=\sqrt{\alpha P_{a}}h_{ac}v+\sqrt{\left(1-\alpha \right)P_{a}}h_{ac}u+\sqrt{\phi P_{c}}h_{cc}z+n_{c},$$

where ϕ denotes the cancellation coefficient. Although the AN is known to Carol, it cannot be absolutely cancelled and, in practice, can be eliminated with a cancellation coefficient, ϕ, where 0<ϕ≤1 [30]. A low value of cancellation coefficient ϕ indicates that the self-interference has nearly been cancelled (ϕ→0: total cancellation; ϕ=1: no cancellation). Since Carol does not know the presence of u, the capacity of v considering u as noise is given by

(31) $$I\left(v;y_{c}\right)=\log_{2}\left(1+\frac{\alpha |h_{ac}{|}^{2}P_{a}}{\sigma_{n}^{2}+\left(1-\alpha \right)|h_{ac}{|}^{2}P_{a}+\phi {\left|h_{cc}\right|}^{2}P_{c}}\right).$$

Alice considers transmitting the overt message at a fixed rate $R_{v}$ due to unknown $h_{ac}$. Since $|h_{ac}{|}^{2}$ has an exponential distribution with a mean of $\sigma_{ac}^{2}$ with its cumulative distribution function (CDF) of

(32) $$\mathrm{Pr}(|h_{ac}{|}^{2}<x)=1-\exp\left(-x/\sigma_{ac}^{2}\right)$$

and $|h_{cc}{|}^{2}$ has an exponential distribution with a mean of $\sigma_{cc}^{2}$ with its probability density function (PDF) of

(33) $$p_{|h_{cc}{|}^{2}}\left(x\right)=\exp\left(-x/\sigma_{cc}^{2}\right)/\sigma_{cc}^{2},$$

the probability of overt decoding the outage probability is obtained by

(34) $$\begin{array}{ccc} P_{\mathrm{out},C}\; & \;=\; & \;\mathrm{Pr}(I\left(v;y_{c}\right)<R_{v})\\ \; & \;=\; & \;\mathrm{Pr}\left(|h_{ac}{|}^{2}<\frac{\left(2^{R_{v}}-1\right)\left(\sigma_{n}^{2}+\phi {\left|h_{cc}\right|}^{2}P_{c}\right)}{\left(\alpha -\left(1-\alpha \right)\left(2^{R_{v}}-1\right)\right)P_{a}}\right)\\ \; & \;=\; & \;1-\int_{0}^{\infty }\exp\left(-\frac{\left(2^{R_{v}}-1\right)\left(\sigma_{n}^{2}+x\phi P_{c}\right)}{{\left(\alpha -\left(1-\alpha \right)\left(2^{R_{v}}-1\right)\right)}^{+}\sigma_{ac}^{2}P_{a}}\right)p_{|h_{cc}{|}^{2}}\left(x\right)dx\\ \; & \;=\; & \;1-\frac{\exp\left(-\frac{\left(2^{R_{v}}-1\right)\sigma_{n}^{2}}{{\left(\alpha -\left(1-\alpha \right)\left(2^{R_{v}}-1\right)\right)}^{+}P_{a}\sigma_{ac}^{2}}\right)}{1+\frac{\left(2^{R_{v}}-1\right)\phi P_{c}\sigma_{cc}^{2}}{{\left(\alpha -\left(1-\alpha \right)\left(2^{R_{v}}-1\right)\right)}^{+}P_{a}\sigma_{ac}^{2}}}. \end{array}$$

The throughput (bits/Hz/s) of the overt message is given by

(35) $$\eta_{v}=R_{v}\times \left(1-P_{\mathrm{out},C}\right).$$

To maximize the throughput of the overt message, the overt transmission rate $R_{v}$ should be chosen properly by numerical search. When α=1 (no transmission of covert message), the overt throughput, denoted as $\eta_{v,nc}$, can be obtained from (34) and (35),

(36) $$\begin{array}{ccc} \eta_{v,nc}\; & \;=\; & \;\max_{R_{v}}R_{v}\times \frac{\exp\left(-\left(2^{R_{v}}-1\right)\sigma_{n}^{2}/\left(P_{a}\sigma_{ac}^{2}\right)\right)}{1+\left(2^{R_{v}}-1\right)\phi P_{c}\sigma_{cc}^{2}/\left(P_{a}\sigma_{ac}^{2}\right)}. \end{array}$$

Therefore, we obtain from (35) and (36) that the overt throughput loss (bits/Hz/s) is given by

(37) $$\eta_{v,\mathrm{loss}}=\eta_{v,nc}-\eta_{v},$$

which can be found by numerical search.

## 6. Numerical Results

The numerical results of the average detection error probability, ξ, the covert throughput, $\eta_{u}$, and the overt throughput loss, $\eta_{v,\mathrm{loss}}$, under different values of the transmit SNR $P_{a}/\sigma_{n}^{2}$, the AN transmit SNR $P_{c}/\sigma_{n}^{2}$ and the cancellation coefficient ϕ are shown. For simplicity, we set $\sigma_{ij}^{2}=1$ for all i,j and, for any figure with a different value of $\sigma_{ij}^{2}$, it will be mentioned. Note that, throughout the simulation, the transmission rate $R_{v}$ used to maximize the overt throughput $\eta_{v}$ in (36) was firstly calculated and then used to compute the average detection error probability and the covert throughput.

### 6.1. Average Detection Error Probability at Warden Willie

**Figure 4:**
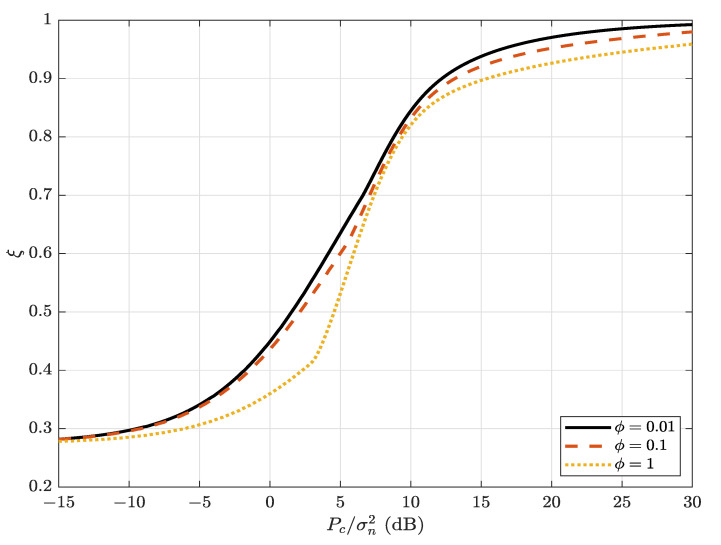
The average detection error probability, ξ, versus Carol’s AN-transmit SNR, $P_{c}/\sigma_{n}^{2}$, for different values of ϕ; α=0.8 and $P_{a}/\sigma_{n}^{2}=5$ dB.

Figure 3 illustrates the average detection error probability ξ versus $P_{a}/\sigma_{n}^{2}$ for different values of the AN transmit SNR, $P_{c}/\sigma_{n}^{2}$. It can be observed that the average detection error probability ξ decreases and converges to 0 as Alice’s transmit power increases. The average detection error probability significantly increases as the AN transmit SNR, $P_{c}/\sigma_{n}^{2}$, increases, even if Willie perfectly knows the AN power, and converges to 1 for a high AN transmit SNR, as also shown in Figure 4 of ξ versus $P_{c}/\sigma_{n}^{2}$ for different values of ϕ. This indicates that the AN helps to improve the covertness because the warden fails to decode the covert message u when the AN power increases. It can also be observed that the warden’s uncertainty (imperfect knowledge) in the received AN power can significantly increases the detection error (for example, a comparison between the line at $P_{c}/\sigma_{n}^{2}=0$ dB and that with perfect knowledge of the received AN power) and, thus, the mutual impact of decoding and AN uncertainty on the warden’s detection error is critical.

Figure 4 illustrates the average detection error probability versus Carol’s AN transmit SNR, $P_{c}/\sigma_{n}^{2}$, for different values of ϕ. It can be observed that the average detection error probability increases as the cancellation coefficient, ϕ, decreases. This means that the improved performance of Carol’s self-interference cancellation can also help to improve the covertness.

**Figure 5:**
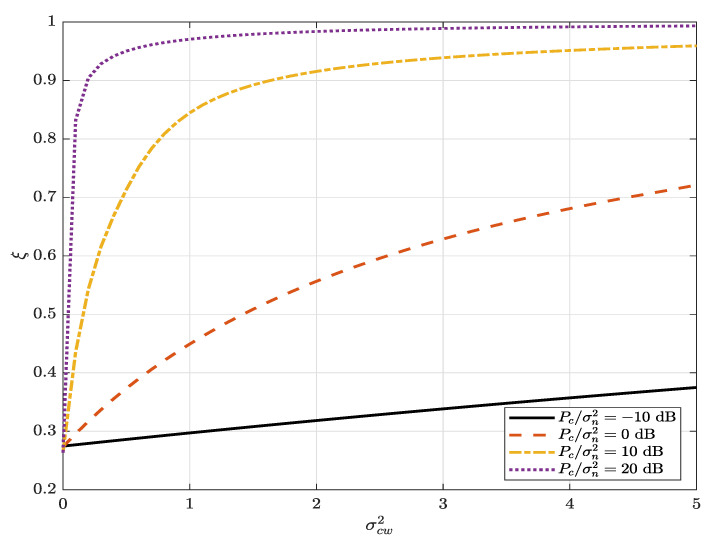
The average detection error probability, ξ, versus $\sigma_{cw}^{2}$ for different values of $P_{c}/\sigma_{n}^{2}$; α=0.8, ϕ=0.01 and $P_{a}/\sigma_{n}^{2}=5$ dB.

Figure 5 illustrates the average detection error probability, ξ, versus $\sigma_{cw}^{2}$ for different values of $P_{c}/\sigma_{n}^{2}$. It can be observed that the average detection error probability increases as $\sigma_{cw}^{2}$ increases (the warden is located close to the overt user) and that the increase is more significant for larger $P_{c}/\sigma_{n}^{2}$. This indicates the efficiency of the AN generated by the overt user to improve the covertness and also emphasizes that the improvement in the secure connection for the overt user results in an improvement in covertness for the covert user.

### 6.2. Covert Throughput and Overt Throughput Loss

**Figure 6:**
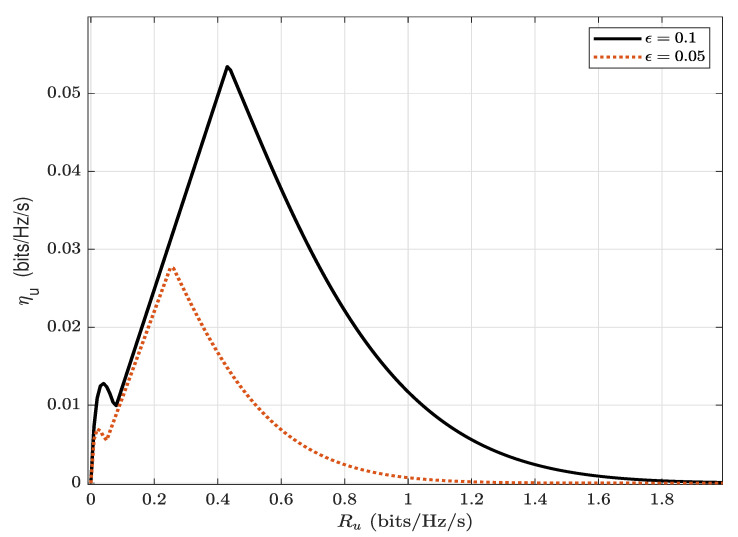
The covert throughput, $\eta_{u}$ (bits/Hz/s), versus $R_{u}$, for different values of ϵ; α=0.8, ϕ=0.01 and $P_{c}/\sigma_{n}^{2}=5$ dB.

Figure 6 presents the covert throughput versus $R_{u}$ (bits/Hz/s) for different values of ϵ. It can be observed that there exists a unique transmission rate of the covert message to maximize the covert throughput; for example, regarding the covertness requirement of 0.1 (ϵ=0.1), the covert throughput is maximized at $R_{u}≃0.42$. It can also be observed that the maximum covert throughput decreases significantly for a stricter covert requirement (ϵ decreases). For example, the maximum covert throughput of 0.028 (bits/Hz/s) for ϵ=0.05 is increased to 0.053 (bits/Hz/s) for ϵ=0.1.

**Figure 7:**
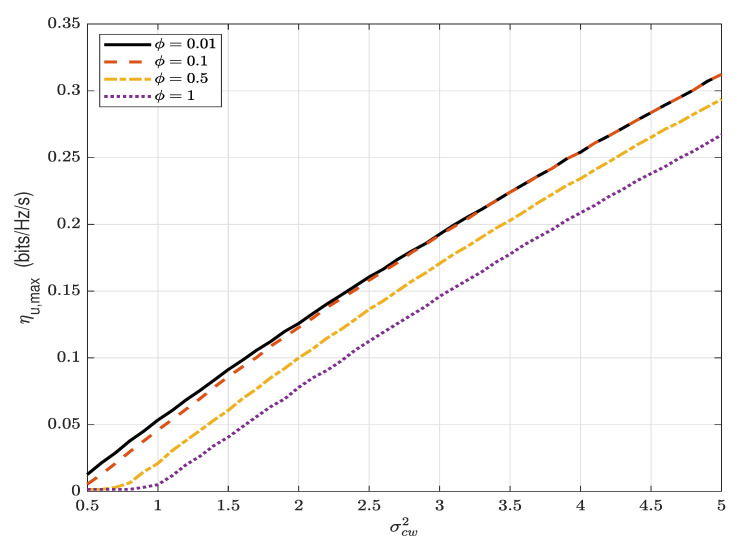
The maximum covert throughput, $\eta_{u,\max}$ (bits/Hz/s), versus $\sigma_{cw}^{2}$, for different values of ϕ; ϵ=0.1, α=0.8 and $P_{c}/\sigma_{n}^{2}=5$ dB.

Figure 7 presents the maximum covert throughput $\eta_{u,\max}$ versus $\sigma_{cw}^{2}$ for different values of ϕ. It can be observed that $\eta_{u,\max}$ increases significantly as $\sigma_{cw}^{2}$ increases and, thus, the AN will be more effective when Willie is located closer to Carol. It can also be observed that the covert throughput increases as ϕ decreases and the increase provided by the better performance of the self-interference cancellation is nearly constant. This indicates the positive impact of the overt receiver’s performance of self-interference cancellation on the covert performance.

**Figure 8:**
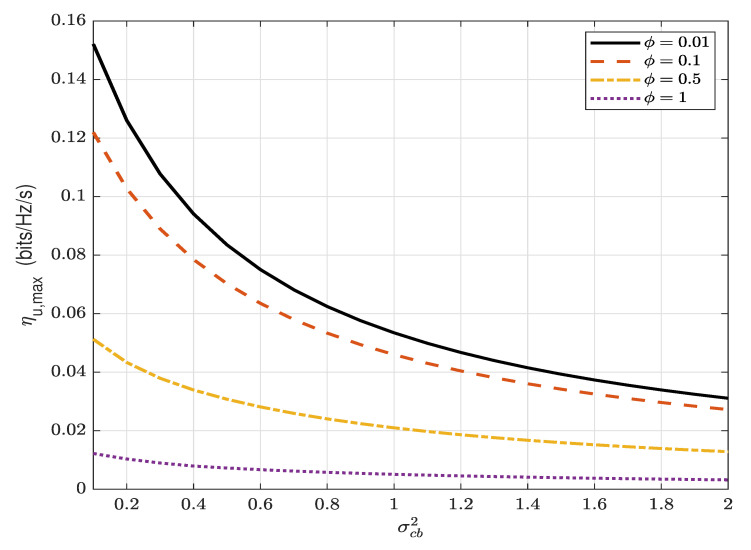
The maximum covert throughput, $\eta_{u,\max}$ (bits/Hz/s), versus $\sigma_{cb}^{2}$, for different values of ϕ; ϵ=0.1, α=0.8 and $P_{c}/\sigma_{n}^{2}=5$ dB.

Figure 8 presents the maximum covert throughput $\eta_{u,\max}$ versus $\sigma_{cb}^{2}$ for different values of ϕ. It can be observed that $\eta_{u,\max}$ decreases significantly as $\sigma_{cb}^{2}$ increases and, thus, the AN makes more interference when Bob is located closer to Carol. It can also be observed that the maximum covert throughput increases as ϕ decreases and the increase is more significant for smaller $\sigma_{cb}^{2}$. This indicates the mutual positive impact of the overt receiver’s performance of self-interference cancellation and the location between Bob and Carol on the covert performance.

**Figure 9:**
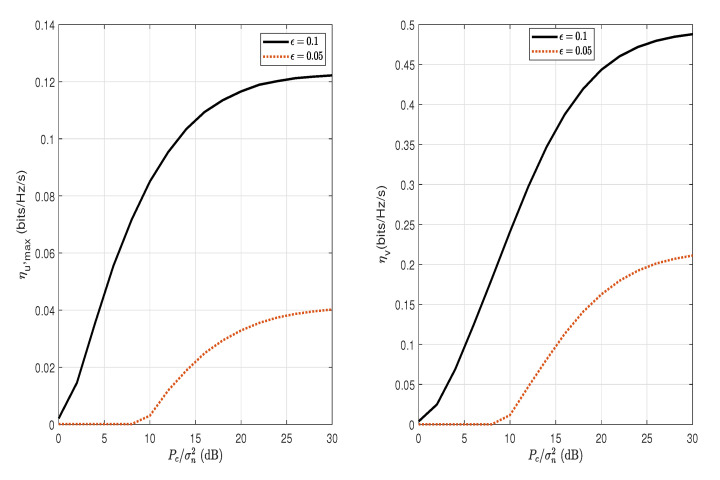
The maximum covert throughput, $\eta_{u,\max}$ (bits/Hz/s), and the overt throughput, $\eta_{v}$, versus the AN transmit SNR, $P_{c}/\sigma_{n}^{2}$, for different values of ϵ; ϕ=0.1 and α=0.8.

**Figure 10:**
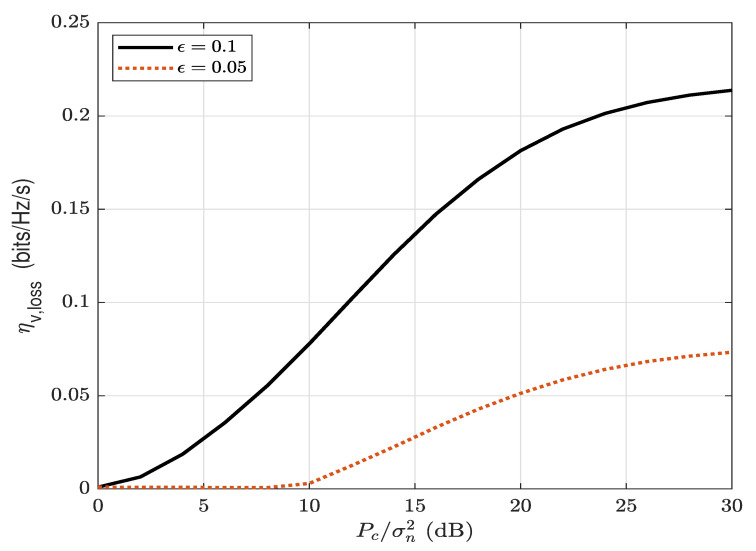
The overt throughput loss, $\eta_{v,\mathrm{loss}}$ (bits/Hz/s), versus the AN transmit SNR, $P_{c}/\sigma_{n}^{2}$, for different values of ϵ; ϕ=0.1 and α=0.8.

Figure 9 and Figure 10 show the maximum covert throughput $\eta_{u,\max}$ with the overt throughput, $\eta_{v}$, and the overt throughput loss $\eta_{v,loss}$ versus the AN transmit SNR $P_{c}/\sigma_{n}^{2}$, respectively, for different values of ϵ. It can be observed in Figure 9 that the covert throughput as well as the overt throughput increases as the transmit AN power, $P_{c}/\sigma_{n}^{2}$, increases. The increase in the covert and overt throughput is due to the increase in the maximum allowed transmission power $P_{a}^{*}$. However, in Figure 10, the overt throughput loss also increases significantly as the AN transmit SNR, $P_{c}/\sigma_{n}^{2}$, increases. This means that, in order to achieve a significant increase in the covert throughput by increasing AN’s transmit power, it requires a trade of high overt throughput loss. For example, regarding $P_{c}/\sigma_{n}^{2}=30$ (dB) and ϵ=0.1, in order to obtain 0.12 (bits/Hz/s) of covert throughput, it requires an overt throughput loss of 0.22 (bits/Hz/s), which is higher than the covert throughput. It can be observed in both Figure 9 and Figure 10 that the increase in covert throughput and overt throughput loss is less significant for stricter covert requirements (smaller ϵ).

In summary, the highlighted results are summarized in Table 2.

## 7. Conclusions and Discussion

This paper exploited the secure connection transmissions with a FD receiver to hide the covert information. The warden’s uncertainties in decoding the overt message and AN-received power were used to guarantee the covertness. The average detection error probability, the covert throughput and the overt throughput loss were calculated. The results showed that AN generated by the overt user can help to improve the covertness and increase the maximum allowed transmit power, and, hence, the covert throughput. The covertness was further improved for the larger transmit power of AN; however, it requires the trade of high overt throughput loss. The result also showed that the improved performance of self-interference cancellation and secure connection at the overt receiver can help to improve the covertness, indicating the positive impact of the improved existing transmissions on the covert performance. In practice, FD communication is still understudied due to its hardware limitations and the requirement of modifying or creating/updating to a new protocol [31]. Consequently, our proposed scheme also has the potential to be implemented and tested on a hardware platform for civil or military applications, and the implementation can be more or less costly, depending on the techniques chosen and the scale of application.

## Figures and Tables

**Table 1:** Table of parameter and metric notations.

Parameter	Notation
Alice’s transmit power	$P_{a}$
Carol’s transmit power or the transmit AN power	$P_{c}$
The power allocation ratio	α
The cancellation coefficient at Carol	ϕ
The channel gain between nodes *i* and *j*	$h_{ij}$
The channel gain variance between nodes *i* and *j*	$\sigma_{ij}^{2}$
The background noise variance	$\sigma_{n}^{2}$
The transmission rate of the overt message, v	$R_{v}$
The transmission rate of the covert message, u	$R_{u}$
Covertness requirement	ϵ
Average detection error probability	ξ
Covert throughput	$\eta_{u}$
Maximum covert throughput	$\eta_{u,\max}$
Overt throughput	$\eta_{v}$
Overt throughput loss	$\eta_{v,\mathrm{loss}}$
Received signal at Bob	$y_{b}$
Received signal at Carol	$y_{c}$
Received signal at Willie	$y_{w}$

**Table 2:** Summary of key results.

	Pros	Cons
Average detection error probability	- increases when the AN power $P_{C}$ increases - increases when the self-interference cancellation is improved (the cancel coefficient ϕ decreases) - increases when the warden is located close to the overt user ($\sigma_{cw}^{2}$ increases)	
Covert throughput	- increases when the AN power $P_{C}$ increases - increases when the self-interference cancellation is improved (the cancel coefficient ϕ decreases) - increases when the warden is located close to the overt user ($\sigma_{cw}^{2}$ increases)	- decreases when the covert user is located close to the overt user ($\sigma_{cb}^{2}$ increases)
Overt throughput loss		- high loss to increase the covert throughput - increases when ϵ increases

## Data Availability

Not applicable.

## Appendix A

In this Appendix, we prove (6). Since $v_{i}$, 1≤i≤n, is identical and has a complex normal distribution with a PDF of

(A1) $$f\left(v_{i}|H_{0}\right)=\exp(-|v_{i}{|}^{2})/\pi .$$

Then, we obtain from (5) that

(A2) $$\begin{array}{ccc} E_{v}\left[\mathrm{Pr}(y_{w}|v,H_{0})\right]\; & \;=\; & \;E_{v}\left[\frac{\exp\left(-\frac{\left|\right|y_{w}-h_{aw}\sqrt{P_{a}}v{\left|\right|}^{2}}{\sigma_{n}^{2}+{\left|h_{cw}\right|}^{2}P_{c}}\right)}{(\pi (\sigma_{n}^{2}+|h_{cw}{|}^{2}P_{c}{))}^{n}}\right] \end{array}$$

(A3) $$\begin{array}{ccc} \; & \;=\; & \;\frac{\prod_{i=1}^{n}\int_{v_{i}}\exp\left(-\frac{|y_{w,i}-h_{aw}\sqrt{P_{a}}v_{i}{|}^{2}}{\sigma_{n}^{2}+{\left|h_{cw}\right|}^{2}P_{c}}\right)\exp(-|v_{i}{|}^{2})dv_{i}}{(\pi^{2}(\sigma_{n}^{2}+|h_{cw}{|}^{2}P_{c}{))}^{n}}. \end{array}$$

Let $h_{aw}=\left|h_{aw}\right|e^{j\rho }$ and ${\tilde{v}}_{i}=v_{i}e^{j\rho }$. Then, the PDF of ${\tilde{v}}_{i}$ is identical to (A1) and, hence, we obtain

(A4) $$\begin{array}{ccc} E_{v}\left[\mathrm{Pr}(y_{w}|v,H_{0})\right]\; & \;=\; & \;\frac{\prod_{i=1}^{n}\int_{v_{i}}\exp\left(-\frac{|y_{w,i}-|h_{aw}{\left|\sqrt{P_{a}}{\tilde{v}}_{i}\right|}^{2}}{\sigma_{n}^{2}+{\left|h_{cw}\right|}^{2}P_{c}}\right)\exp(-|{\tilde{v}}_{i}{|}^{2})dv_{i}}{(\pi^{2}(\sigma_{n}^{2}+|h_{cw}{|}^{2}P_{c}{))}^{n}}. \end{array}$$

For real values of *t* and *z*, one can show that

(A5) $$\int_{-\infty }^{\infty }\exp(-(t-|h_{aw}{|}^{2}\sqrt{P_{a}}{z)}^{2}/a)\exp\left(-z\right)dz=\sqrt{\frac{\pi aP_{a}}{a+|h_{aw}{|}^{2}P_{a}}}\exp(-t/(a+|h_{aw}{|}^{2}P_{a})).$$

Then, it can be obtained from (A4) that

(A6) $$\begin{array}{ccc} E_{v}\left[\mathrm{Pr}(y_{w}|v,H_{0})\right]\; & \;=\; & \;\frac{\prod_{i=1}^{n}\exp\left(-\frac{|y_{w,i}{|}^{2}}{\sigma_{n}^{2}+|h_{cw}{|}^{2}P_{c}+{\left|h_{aw}\right|}^{2}P_{a}}\right)}{(\pi (\sigma_{n}^{2}+|h_{cw}{|}^{2}P_{c}+|h_{aw}{|}^{2}P_{a}{))}^{n}} \end{array}$$

(A7) $$\begin{array}{ccc} \; & \;=\; & \;\frac{\exp\left(-\frac{\left|\right|y_{w}{\left|\right|}^{2}}{\sigma_{n}^{2}+|h_{cw}{|}^{2}P_{c}+{\left|h_{aw}\right|}^{2}P_{a}}\right)}{(\pi (\sigma_{n}^{2}+|h_{cw}{|}^{2}P_{c}+|h_{aw}{|}^{2}P_{a}{))}^{n}}. \end{array}$$

Similarly,

(A8) $$\begin{array}{ccc} E_{v}\left[\mathrm{Pr}(y_{w}|v,H_{1})\right]\; & \;=\; & \;\frac{\prod_{i=1}^{n}\int_{v_{i}}\exp\left(-\frac{|y_{w,i}-h_{aw}\sqrt{\alpha P_{a}}v_{i}{|}^{2}}{\sigma_{n}^{2}+|h_{cw}{|}^{2}P_{c}+\left(1-\alpha \right){\left|h_{aw}\right|}^{2}P_{a}}\right)\exp(-|v_{i}{|}^{2})dv_{i}}{(\pi^{2}(\sigma_{n}^{2}+|h_{cw}{|}^{2}P_{c}+\left(1-\alpha \right)|h_{aw}{|}^{2}P_{a}{))}^{n}} \end{array}$$

(A9) $$\begin{array}{ccc} \; & \;=\; & \;\frac{\exp\left(-\frac{\left|\right|y_{w}{\left|\right|}^{2}}{\sigma_{n}^{2}+|h_{cw}{|}^{2}P_{c}+{\left|h_{aw}\right|}^{2}P_{a}}\right)}{(\pi (\sigma_{n}^{2}+|h_{cw}{|}^{2}P_{c}+|h_{aw}{|}^{2}P_{a}{))}^{n}}, \end{array}$$

where (A9) is also derived following (A5).

## Appendix B

In this Appendix, we find the optimal detection threshold used to minimize $\left(P_{f}^{\prime }\left(h_{aw}\right)+P_{m}^{\prime }\left(h_{aw}\right)\right)$ and its resulting minimum. Let $x=1/\lambda^{\prime }$. Since $r_{1}\geq r_{0}$, $s_{0}\geq s_{1}$, $\delta_{0}\geq \delta_{1}$, it follows from (14) and (15) that

(A10) $$\begin{array}{ccc} \; & & \;P_{f}^{\prime }\left(h_{aw}\right)+P_{m}^{\prime }\left(h_{aw}\right)\\ \; & \;=\; & \;\left\{\;\begin{array}{cc} 1-\exp\left(-\delta_{0}\right),\;x\geq \frac{s_{0}+\delta_{0}}{r_{0}}, & \\ 1-\exp\left(-\left(r_{0}x-s_{0}\right)\right),\;\frac{s_{1}+\delta_{1}}{r_{1}}\leq x<\frac{s_{0}+\delta_{0}}{r_{0}}, & \\ 1-\exp\left(-\left(r_{0}x-s_{0}\right)\right)+\exp\left(-\left(r_{1}x-s_{1}\right)\right)-\exp\left(-\delta_{1}\right),\;x<\frac{s_{1}+\delta_{1}}{r_{1}}. \end{array}\right. \end{array}$$

It follows from the second function of (A10) that $\left(P_{f}^{\prime }\left(h_{aw}\right)+P_{m}^{\prime }\left(h_{aw}\right)\right)$ is an increasing function of *x* for $\left(s_{1}+\delta_{1}\right)/r_{1}\leq x<\left(s_{0}+\delta_{0}\right)/r_{0}$ and, hence, $\left(P_{f}^{\prime }\left(h_{aw}\right)+P_{m}^{\prime }\left(h_{aw}\right)\right)$ is minimized at $x=\left(s_{1}+\delta_{1}\right)/r_{1}$ and maximized at $x=\left(s_{0}+\delta_{0}\right)/r_{0}$. Then, the minimum of $\left(P_{f}^{\prime }\left(h_{aw}\right)+P_{m}^{\prime }\left(h_{aw}\right)\right)$ can be found by finding the optimum detection threshold and minimum of

(A11) $$\begin{array}{ccc} P_{f}^{\prime }\left(h_{aw}\right)+P_{m}^{\prime }\left(h_{aw}\right)\; & \;=\; & \;1-\exp\left(s_{0}-r_{0}x\right)+\exp\left(s_{1}-r_{1}x\right)-\exp\left(-\delta_{1}\right), \end{array}$$

for $x<\left(s_{1}+\delta_{1}\right)/r_{1}$. Taking the first derivative and setting (A11) to zero yields

(A12) $$x=\min\{\left(\ln\left(r_{1}/r_{0}\right)-\left(s_{0}-s_{1}\right)\right)/\left(r_{1}-r_{0}\right),\left(s_{1}+\delta_{1}\right)/r_{1}\}$$

and, then, the resulting minimum of $\left(P_{f}^{\prime }\left(h_{aw}\right)+P_{m}^{\prime }\left(h_{aw}\right)\right)$.
